# Depression and unemployment incidence rate evolution in Portugal, 1995–2013: General Practitioner Sentinel Network data

**DOI:** 10.11606/S1518-8787.2017051006675

**Published:** 2017-11-07

**Authors:** Ana Paula Rodrigues, Mafalda Sousa-Uva, Rita Fonseca, Sara Marques, Nuno Pina, Carlos Matias-Dias

**Affiliations:** I Instituto Nacional de Saúde Doutor Ricardo Jorge. Departamento de Epidemiologia. Lisboa, Portugal; II Universidade Nova de Lisboa. Centro de Investigação em Saúde Pública. Lisboa, Portugal; III Agrupamento de Centros de Saúde Dão-Lafões. Unidade Cuidados de Saúde Personalizados Tomaz Ribeiro. Tondela, Portugal; IV Agrupamento de Centros de Saúde Dão-Lafões. Unidade de Saúde Familiar Rio Dão. Santa Comba-Dão, Portugal

**Keywords:** Depression, epidemiology, Unemployment, Socioeconomic Factors, Ecological Studies, Depressão, epidemiologia, Desemprego, Fatores Socioeconómicos, Estudos Ecológicos

## Abstract

**OBJECTIVE:**

Quantify, for both genders, the correlation between the depression incidence rate and the unemployment rate in Portugal between 1995 and 2013.

**METHODS:**

An ecological study was developed to correlate the evolution of the depression incidence rates estimated by the General Practitioner Sentinel Network and the annual unemployment rates provided by the National Statistical Institute in official publications.

**RESULTS:**

There was a positive correlation between the depression incidence rate and the unemployment rate in Portugal, which was significant only for males (R^2^ = 0.83, p = 0.04). For this gender, an increase of 37 new cases of depression per 100,000 inhabitants was estimated for each 1% increase in the unemployment rate between 1995 and 2013.

**CONCLUSIONS:**

Although the study design does not allow the establishment of a causal association between unemployment and depression, the results suggest that the evolution of unemployment in Portugal may have had a significant impact on the level of mental health of the Portuguese, especially among men.

## RESUMO

**OBJECTIVO:**

Quantificar, para ambos os sexos, a correlação entre a taxa de incidência de depressão e a taxa de desemprego, em Portugal, entre 1995 e 2013.

**MÉTODOS:**

Foi desenvolvido um estudo ecológico no qual se correlacionou a evolução das taxas de incidência de depressão estimadas pela Rede Médicos Sentinela e as taxas de desemprego anuais disponibilizadas pelo Instituto Nacional de Estatística em publicações oficiais.

**RESULTADOS:**

Observou-se uma correlação positiva entre taxa de incidência de depressão e taxa de desemprego em Portugal, sendo esta significativa apenas para o sexo masculino (R^2^ = 0,83; p = 0,04). Estimou-se, para este sexo, um aumento de 37 novos casos de depressão por 100.000 habitantes a cada 1% de aumento da taxa de desemprego entre 1995 e 2013.

**CONCLUSÕES:**

Embora o desenho do estudo não permita o estabelecimento de uma relação causal entre desemprego e depressão, os resultados obtidos sugerem que a evolução do desemprego em Portugal poderá ter tido um impacto não desprezável no nível de saúde mental dos portugueses, em especial no sexo masculino.

## INTRODUCTION

The international context of the economic and financial crisis has brought political change to the European Region in a short period of time. In Portugal, the international context and the signing of a Memorandum of Understanding on economic policy conditionality between Portugal and the *Troika* in May 2011 imposed, as in other European countries, in particular in southern Europe, the adoption of a set of cross austerity measures to all areas of governance.

Reducing financial burdens on employment protection, the social sector, and health are the measures that have the greatest negative impact on the social determinants of health, including unemployment^4^. In Portugal, the unemployment rate in all age groups and social classes reached its peak in the first quarter of 2013 (17.5% for both genders and 17.7% for males)^a^.

Unemployment, especially prolonged unemployment, has been associated with a change in the health status of individuals and the adoption of less healthy lifestyles, along with less access to health care, as reported by McKee M et al.^21^ This may contribute to increased depression, suicides, addictive behaviors, and altered sleep patterns^21^ ^,^ ^b^.

The hypothesis that unemployment may have a negative effect on mental health (depression, anxiety, psychosomatic symptoms, subjective well-being, and self-esteem) is not new and has been widely studied^6^ ^,^ ^24^ ^,^ ^25^ in previous crisis contexts, and is now one of the main subjects under study in the European Region^9^ ^,^ ^13^ ^,^ ^14^. The negative effect of unemployment on mental health was even higher in countries with lower levels of economic development, unequal distribution of wealth, low investment in social policies, or with high levels of unemployment in the periods immediately preceding the crisis period^5^ ^,^ ^10^ ^,^ ^16^ ^,^ ^25^.

Considering that countries have distinctive characteristics and patterns of adaptation and resilience, the World Health Organization (WHO)^29^ states that the current challenge is to understand and monitor the impact that economic and social crisis may have on the health of populations in each country in particular, in order to identify the most susceptible groups during crisis periods and thus contribute to the implementation of protective strategies directed towards these groups^29^.

According to these WHO recommendations^29^, studies in the Greek population revealed a change in the risk factors for depression during the crisis. In 2008, being female, divorced, or widowed and being unemployed were the main risk factors for depression^19^; however, in 2011, an increased risk of depression was identified in young, married, and unemployed individuals^8^.

Also in Spain, the economic recession appeared to significantly increase mental health problems in primary care users, particularly in families affected by unemployment and with difficulty in paying loans^12^. In Portugal, knowledge of the effects of the current crisis on population health is still scarce^2^.

Data from the Portuguese GP Sentinel Network show an increase in the depression incidence rate in 2012, especially in males aged 55–64 years-old (277.3/100,000 in 2004 and 859.8/100,000 in 2012). These results are favorable to the hypothesis of an increased risk of male depression at times of economic and social crisis, as already described in other southern European countries^1^ ^,^ ^25^. Indeed, in countries with high unemployment rates, men, regardless of their working conditions, have a lower level of mental health (and higher demand for health care), possibly associated with the stigma of male unemployment and job insecurity^5^. However, in females, this effect was only observed in employed women^5^. Although these differences may be partly explained by the gender differences observed in the employment and health binomial, in recent years there has been an increase for both genders in the inequalities of employment between people with and without long-term limitations^20^.

Based on the assumption of an association between unemployment and depression^5–7^, this study aimed to quantify, for each gender, the correlation between the depression incidence rate and the unemployment rate in Portugal in the last few decades, with the hypothesis that this correlation may be stronger in males, given their greater risk of developing mental illness in periods of economic and social crisis^5^. The choice of the unemployment rate was because it is an indicator sensitive to the changes that occurred during periods of crisis, also used in other studies in this context^5^ ^,^ ^26^ ^,^ ^28^.

## METHODS

In the present study, we developed an ecological correlation study using values of the depression incidence rates from the General Practitioner Sentinel Network (GP Sentinel Network) and the annual unemployment rates provided by the National Statistical Institute in official publications between 1995–1997, 2004, and 2012–2013^a^.

The GP Sentinel Network is a health observation system made up of family doctors from the National Health Service who voluntarily notify, in a continuous and systematic manner, several health events that occur in their patients. The population under effective observation in the GP Sentinel Network used to calculate incidence rates results from the sum of the patient lists of active physicians in each week, that is, the physicians who reported cases each week. In the years under study, the population under observation in the Network varied between a minimum of 28,184 individuals in 2013 and a maximum of 164,676 individuals in 1995.

We used the depression incidence rates found in the population under observation by the GP Sentinel Network between the years 1995, 1996, 1997, 2004, 2012, and 2013.

Notification events vary each year; thus, depression was only under study in 1995–1997, 2004, 2012, and 2013, which is why incidence rates are only available for those years. In the 1995–1997 triennium, the reporting events were all the new cases of depression (first episode in the life of the patient or subsequent episodes); in 2004, 2012, and 2013, all the appointments for depression with identification of the reason for the appointment in each case (first episode in the life of the patient, subsequent episodes, follow-up appointment or appointment for medication renewal) were being notified. In all the years under study, the definition of a case of depression was based on the best clinical knowledge, that is, following the same criteria used in the diagnostic and therapeutic approach to the patient, and there is also a manual of procedures to standardize the way cases were notified. Considering only the first episode in the life of the patient or the subsequent episodes, it was possible to calculate the depression incidence rate in a similar way for all the years under study.

Depression rates were standardized through the direct method using the European standard population of 1976. The ratio of incidence rates for both genders was estimated for the years under analysis. Using a linear regression model, the correlation between the unemployment rate and the depression incidence rate was estimated for each gender. The normality of the data (assumption for the application of the linear model) was verified using the Shapiro-Wilk test. We considered a level of statistical significance of 5%.

## RESULTS

There was an increase in the depression incidence rate starting in 2004 for both genders. Females had higher incidence rates in all years; however, the ratio between genders decreased in 2012 and 2013 (Table).

**Table:** Unemployment rate and depression incidence rate in Portugal between 1995 and 2013.

Year	Male			Female			Ratio of standardized depression incidence rates (female/male)
	
	Unemployment rate (/100,000)	Depression incidence rate (/100,000)	Depression incidence rate (standardized) (/100,000)	Unemployment rate (/100,000)	Depression incidence rate (/100,000)	Depression incidence rate (standardized) (/100,000)	
1995	0.063	178.0	181.1	0.081	789.0	0788.2	4.4
1996	0.064	143.3	143.7	0.082	879.9	0882.5	6.1
1997	0.060	172.3	164.7	0.075	841.3	0828.2	5.0
2004	0.058	476.6	451.8	0.076	1.963.9	1.873.3	4.1
2012	0.157	571.5	539.5	0.156	2.136.0	1.968.0	3.6
2013	0.161	731.4	668.3	0.164	2.103.5	1.928.7	2.9

**Figure 1:**
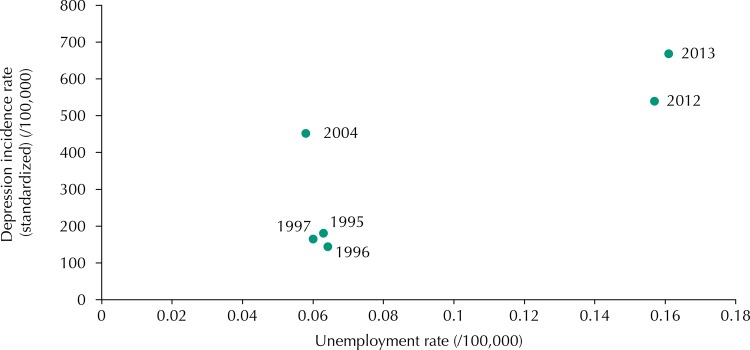
Correlation between unemployment and standardized depression incidence rate in Portugal, for men.

**Figure 2:**
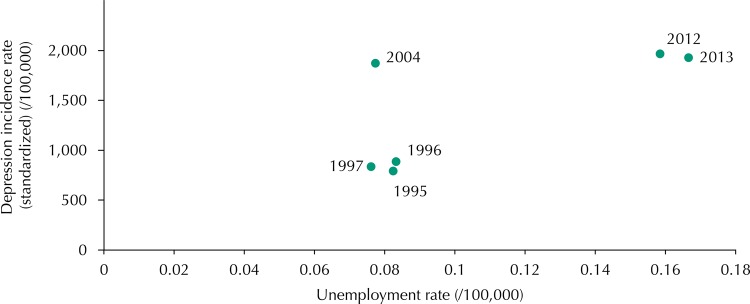
Correlation between unemployment and standardized depression incidence rate in Portugal, for women.

There was a greater correlation between unemployment and depression in males (R^2^ = 0.83, p = 0.04) (Figure 1), in which about 83% of the variability of the depression incidence rate was related to the value of the unemployment rate. In females, the estimated correlation was not statistically significant (R^2^ = 0.71, p = 0.11) (Figure 2).

Considering the equation of the line that shows the relationship between the depression incidence rate and the unemployment rate (depression incidence rate = 3,706.5 × unemployment rate + 10.38), it is possible to estimate an increase of 37 new depression cases per 100,000 inhabitants per 1% increase in the male unemployment rate.

## DISCUSSION

In this study, there was a positive correlation between the depression incidence rate and the unemployment rate in Portugal between 1995 and 2013, which was significant only for males, in the years during which the country was in an economic, financial and social crisis. These results agree with those obtained by other authors^7^ ^,^ ^24^, and also found in other countries during a crisis situation and consequent austerity^1^ ^,^ ^12^ ^,^ ^13^ ^,^ ^2^ ^3^. Given the methodological similarities, it should be noted that a Greek study found a positive correlation between unemployment and suicide in men, but not in women^26^.

In fact, a study by Buffel et al.^5^ showed that, in countries with high unemployment rates, men, regardless of their working conditions, have a lower level of mental health (and higher demand for health care).

Such results have been attributed to the social role and the family responsibilities historically associated with men. For this reason, men may feel more threatened and have more difficulty dealing with the social stigma of unemployment relative to women, which puts them at greater risk of developing mental illnesses^18^ ^,^ ^20^ ^,^ ^25^.

Given that, currently, most individuals with mental health problems rely on family doctors^22^, the use of the depression incidence rate in primary health care is considered a sensitive indicator to analyze this health problem. The use of primary data collected voluntarily by Sentinel doctors obviated the limitation highlighted in current clinical records regarding the need for greater adherence to the electronic registry of these health problems at the primary health care level^22^. Although cases that, due to several factors (social, cultural, or health care accessibility), do not rely on health services are excluded, considering that this effect occurred in all the years under study, the indicator chosen can be understood as a proxy for the increase in depression risk in the community. Despite this, we recognize that relying on the demand for medical care can be influenced by possible changes in health seeking behavior pattern in Portugal. Also, the case definition used (new cases of depression, which use a primary health care appointment, defined according to the best clinical knowledge) does not allow us to distinguish different levels of severity in the presentation of this pathology but reflects the set of situations which justify medical care demand.

The change in the definition of a depression case in the GP Sentinel Network in 2004 (appointments for depression rather than new cases of depression), together with the fact that physicians may be more attentive to this health problem, may have contributed for the reporting of prevalent cases as incidents, leading to an overestimation of the incidence on that year and consequently reducing the observed correlation between unemployment and depression. It should be noted, however, that our results corroborate the increase in the proportion of patients with mental health problems in primary health care between 2011 and 2013 observed by the General Health Directorate^22^ and the increase in antidepressant consumption in Portugal observed between 2000 and 2012^11^.

Also, a Belgian study^3^, conducted by a network of sentinel doctors, estimated a depression incidence rate of 719/100,000 males and 1,440/100,000 females in 2008. These figures, although different from those observed in Portugal in 2012 (571.5/100,000 males, 2,136.0/100,000 females), are within the same order of magnitude, which indicates an adequacy of the methodology used.

We assume, however, that the design of the study (ecological study) does not allow us to establish causal relations between the exposure variable (unemployment) and the outcome variable (depression), so we recognize the need to develop studies with more robust designs that allow the confirmation of the hypothesis pointed out. However, despite the above limitations, this is one of the first studies whose results indicate that, in Portugal, the increase in unemployment may have had a significant impact on the level of mental health of the Portuguese and, consequently, on the demand for health care. It also highlights that the magnitude of this association was different between the two genders, suggesting that during the period under study, the economic and social crisis may have had a more negative impact on males.

Although some theories suggest an increase in depression in modern societies as a result of environmental changes and the social context^15^, the results obtained in the present study, in agreement with similar studies carried out in previous years, and in the current context of economic crisis, suggest the need to strengthen the monitoring of this mental health problem, particularly among males. This monitoring may contribute to the effective implementation of mental illness prevention strategies, directed especially at the most vulnerable groups, identified in the current Portuguese context.

## Footnotes

a Instituto Nacional de Estatística (PRT). Dados estatísticos: principais indicadores. Lisboa; 2015 [cited 2015 Jun 11]. Available from: https://www.ine.pt/xportal/xmain?xpid=INE&xpgid=ine_indicadores&indOcorrCod=0005599&contexto=pi&selTab=tab0

b Dias CM, Fonseca R, Contreiras T, Pereira-Miguel J. Os efeitos da austeridade na saúde da população: evidência internacional e experiência portuguesa. In: Ferreira EP, coordenador. A austeridade cura? A austeridade mata?. Lisboa: Associação Académica da Faculdade de Direito de Lisboa; 2013. p.927-54.
